# Consequences of climate-driven relocation in the Prehispanic US Southwest

**DOI:** 10.1016/j.isci.2026.118133

**Published:** 2026-11-06

**Authors:** S.G. Ortman

**Affiliations:** 1 Department Institute of Behavioral Science, University of Colorado Boulder, Boulder, CO, USA; 2 Santa Fe Institute, Santa Fe, NM, USA; 3 Archaeology Southwest, Tucson, AZ, USA

**Keywords:** climate adaptation, urban relocation, archaeology, US Southwest, migration

## Abstract

As humanity copes with a warming planet, there is a growing recognition that in some situations entire communities may be forced to relocate. This perspective paper summarizes an especially well-understood episode of climate-driven relocation that took place in the Four Corners region of the US Southwest in the 13^th^ century CE. This episode is well understood due to an exceptional archaeological record with high temporal resolution, oral traditions that have been maintained in descendant communities, a long history of research, and significant investment in synthesis of both archaeological and paleoclimatic records. The account is also well established in that it is based on a robust quantity and quality of evidence and high agreement among experts. The account is organized around questions that are often raised regarding community relocation in the present, thus providing an example of how archaeological research can contribute to the knowledge base for successful climate change adaptation responses today.

## Graphical abstract

**Figure:**
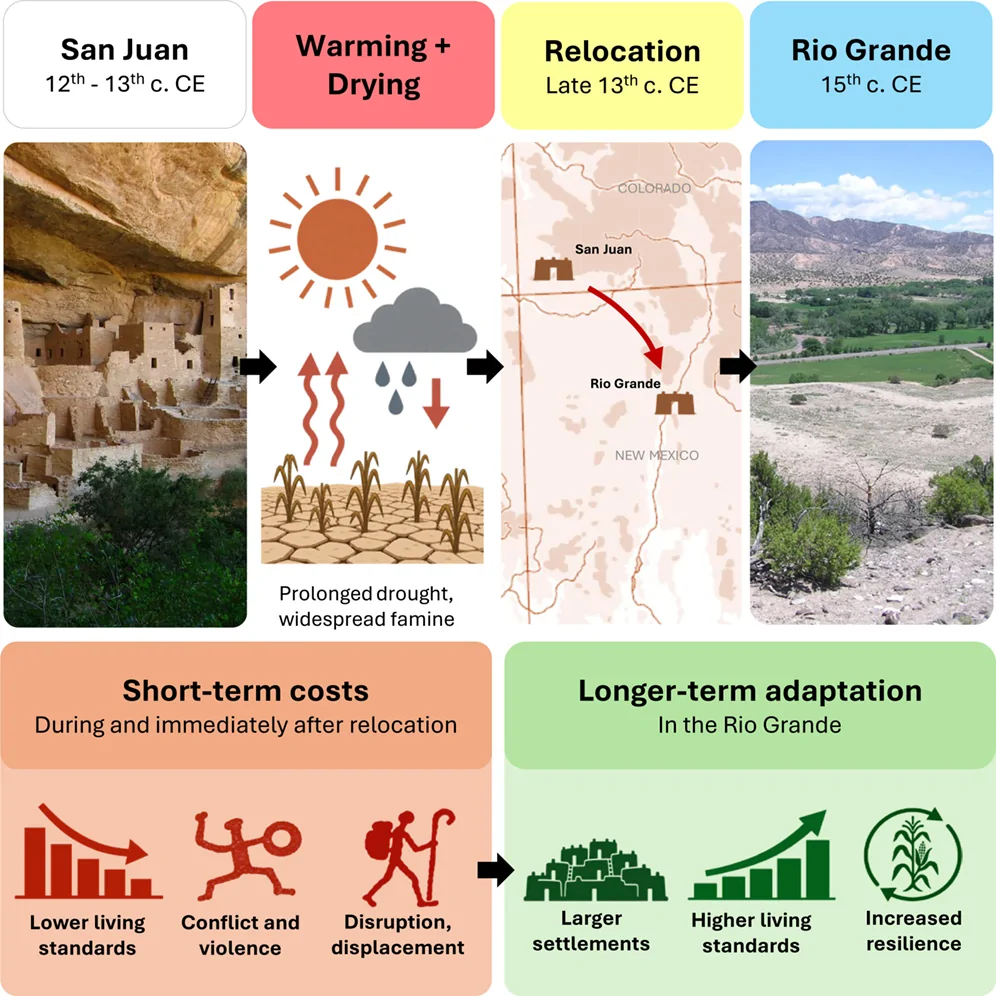

## Teaser

Archeology; Environmental science; Global change; Human geography

## Introduction

As humanity begins to cope with the consequences of a warming planet, there is a growing recognition that in some situations entire communities will be forced to relocate.^1^^,^^2^^,^^3^ The Intergovernmental Panel on Climate Change defines ***urban relocation*** as an ***adaptation response*** to climate change that involves the planned or managed movement of people, assets, and infrastructure away from areas highly exposed to climate risks.^4^^,^^5^^,^^6^ The goal is to reduce vulnerability and enhance adaptive capacity. Relocation is viewed as a last-resort adaptation when in-place solutions are insufficient or not feasible. Certain urban conditions and geographical locations make it more likely. Systems that are highly exposed to climate hazards, have limited capacity to adapt in place, or face irreversible environmental changes are the most likely candidates for relocation. Also, unlike migration, urban relocation is seen as government-led and supported by policies aimed at reducing risks from climate hazards.

Climate-driven urban relocation has been infrequent in recent times, and this can lead one to view the challenges associated with relocation as unprecedented. However, if one adopts a longer view, the archaeological record provides many examples of relocation, whether of ancient cities or smaller towns and villages, driven by a variety of social, political, and economic forces, including climate change. There is thus a need for experts on various episodes of relocation in history to summarize what is known about specific cases to build a knowledge base for climate change adaptation in the present. The purpose of this perspective is to summarize what is known about one especially well-studied case of large-scale human relocation that took place seven centuries ago, in what is now the Four Corners region of the US Southwest. Although this society was not urbanized by modern definitions, the settlements that relocated included the largest settlements of that society, and urban science has shown that the largest settlements in every society exhibit most of the same dynamics and respond to the same factors that influence contemporary cities (see the Discussion for more details).^7^^,^^8^^,^^9^^,^^10^^,^^11^ Also, the relocation episode involved the planned movement of people and infrastructure away from an area exposed to climate-related hazards, where opportunities to adapt in place were limited by the prior history of development of the society. There is evidence that this relocation was driven by political leadership and by policies that aimed to reduce risks from climate hazards by relocating to less vulnerable locations. Finally, the relocated population integrated into an existing population in the new area.

There is a long and rich tradition of research that investigates relationships between climate and migration in the US Southwest, at scales ranging from specific canyon systems to the entire region.^12^^,^^13^^,^^14^^,^^15^^,^^16^^,^^17^^,^^18^ Indeed, climate-related mobility has been a major focus of research in US Southwest archaeology ever since A.E. Douglas first used tree rings to date archaeological sites and reconstruct the associated weather patterns, and a huge literature that addresses climate-driven mobility, across many cases and many times and places, has accumulated.^12^^,^^15^^,^^19^^,^^20^^,^^21^^,^^22^^,^^23^^,^^24^^,^^25^^,^^26^^,^^27^ Here, I focus on the best-known and best-documented regional case. The relocation of several tens of thousands of people from the northern drainages of the San Juan River to the Northern Rio Grande Valley in the late 1200s CE is well understood due to an exceptional archaeological record with high temporal resolution, oral traditions that have been maintained in descendant communities, a long history of research, and significant investment in synthesis of both archaeological and paleoclimatic records.^28^^,^^29^^,^^30^^,^^31^^,^^32^^,^^33^^,^^34^^,^^35^ Although not all experts agree on all details, the account offered here is well established in that it is based on a robust quantity and quality of evidence and high agreement among experts.^36^

The account is organized to address several questions that are often raised regarding relocation as a climate change adaptation response in the present. What were the social processes involved? Who decided to relocate? How did it take place? How rapidly did it occur? Was it coerced or voluntary, violent or peaceful? What were the consequences for living standards? Did the relocated population prosper or struggle? What we shall see is that the answers to these questions depend on the timescale over which they are assessed. The relocation began slowly but accelerated as it progressed; it began as a migration flow, but it became politically organized as it proceeded; it was initially peaceful but became violent; and the population initially suffered but was much better off in the long run. These complications suggest the consequences of climate adaptation alternatives in contemporary policy discussions would also benefit from a multi-temporal scale of assessment.

## The relocation stage

The episode of relocation discussed here concerns the Ancestral Puebloan society that took root in the northern drainages of the San Juan River, in present-day Colorado and Utah, around 600 CE (Figure 1). The people of this society were maize horticulturalists who relied upon deep loess soils of upland plateaus ranging from 1,600 to 2,400m in elevation, and at approximately 37 degrees North latitude. Precipitation in this region was bimodal, consisting of winter snows that percolated into the soil, and summer monsoons that replenished this moisture in the months of July and August. Tree-ring records and pollen cores provide a strong basis for reconstructing paleoclimatic conditions, which show that annual precipitation and temperature both changed, somewhat independently, across the period of occupation.^37^^,^^38^

**Figure 1:**
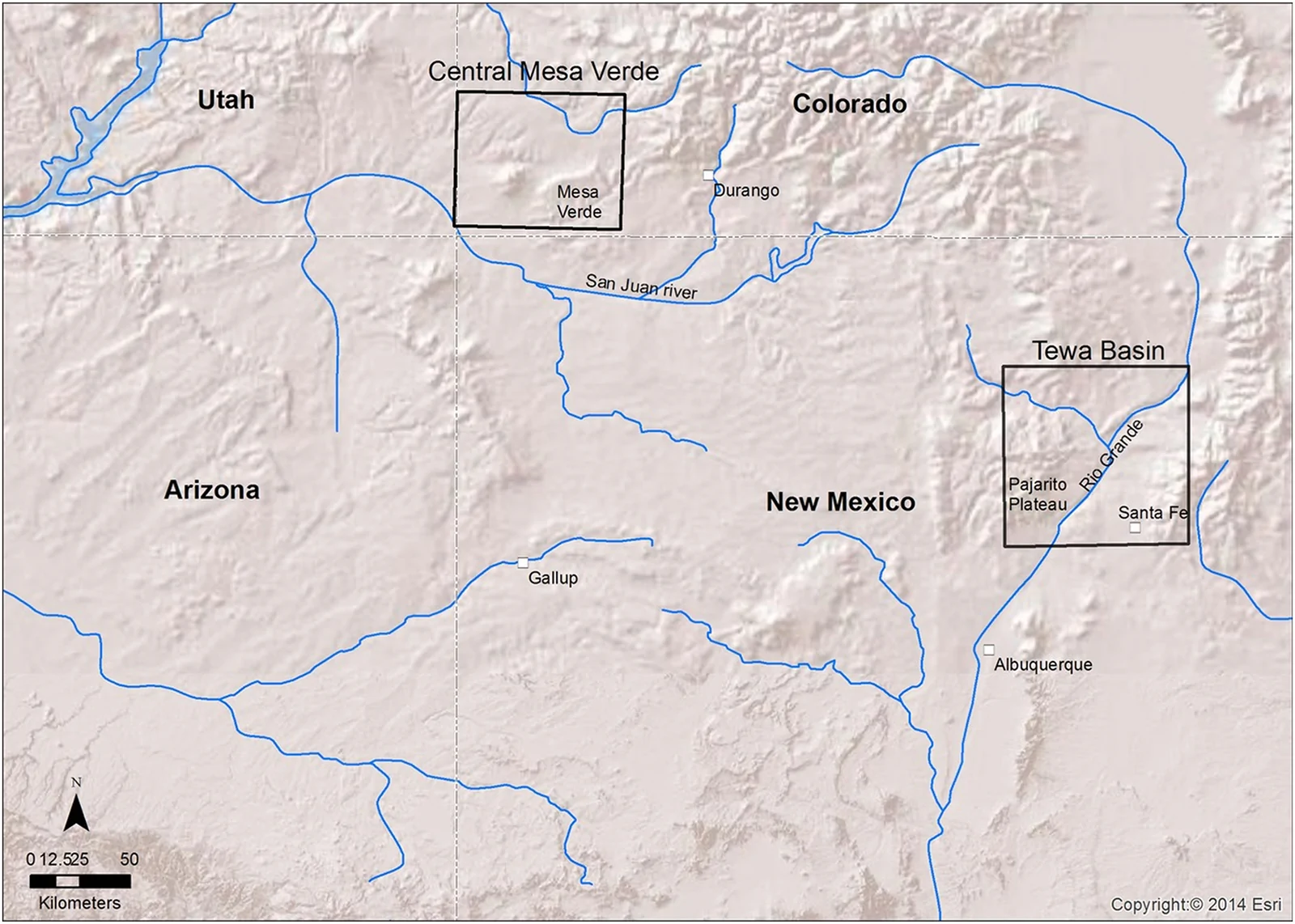
The US Southwest with focal pre- and post-relocation study areas

Figure 2 summarizes the current, tree-ring-based paleoenvironmental reconstruction for the 4,600km^2^ rectangular area labeled “Central Mesa Verde” in Figure 1. The data are extracted from the PaleoCAR application (https://www.openskope.org/paleoenvironmental-reconstruction-paleocar) on the SKOPE website.^31^ The reconstruction is based on correlation-adjusted regression of tree-ring series with 100+ years of contemporary climate data modeled by PRISM at an 800-m scale resolution to retrodict water-year precipitation (A) and maize growing degree days (B) for each year between 600 and 1300 CE. The mean value across all 800-m pixels comprising the Central Mesa Verde study area is shown, as is a centered 11-year running mean. The chart shows that the residents of this area experienced significant fluctuations in climate over the centuries, including extended periods of notably dry and warm conditions, such as 900–940 CE, 1130–1160 CE, 1220–1260 CE, and 1275–1290 CE. These charts also show that people experienced significant interannual variability in climate, which may have made it difficult for farmers to discern longer-term trends.

**Figure 2:**
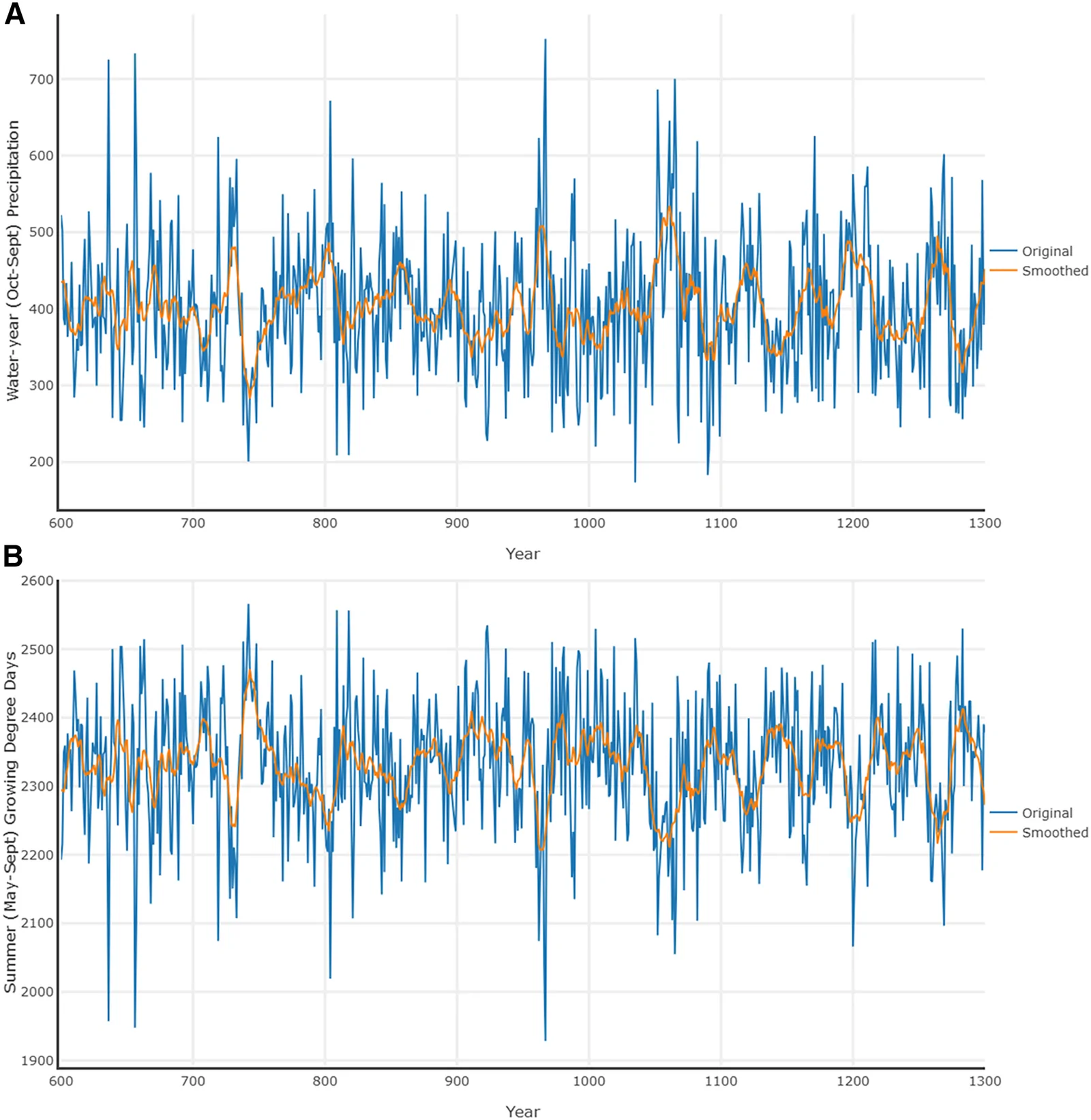
A, retrodicted water-year precipitation, and B, maize growing degree days, during the Ancestral Pueblo occupation of the Central Mesa Verde Region The data are extracted from https://www.openskope.org/paleoenvironmental-reconstruction-paleocar.

A separate annual mean temperature reconstruction derived from the Neotoma pollen database (https://www.neotomadb.org/), which covers a broader area and longer time period, is given in Figure 3.^39^^,^^40^ The deviations here are in °C relative to the 1961–1990 CE baseline. This reconstruction shows that annual mean temperatures were generally cooler than those today for most of the Ancestral Pueblo occupation, but there was a marked increase of nearly 1°C between 950 and 1150 CE, and these elevated temperatures persisted through the end of the Ancestral Pueblo occupation.

**Figure 3:**
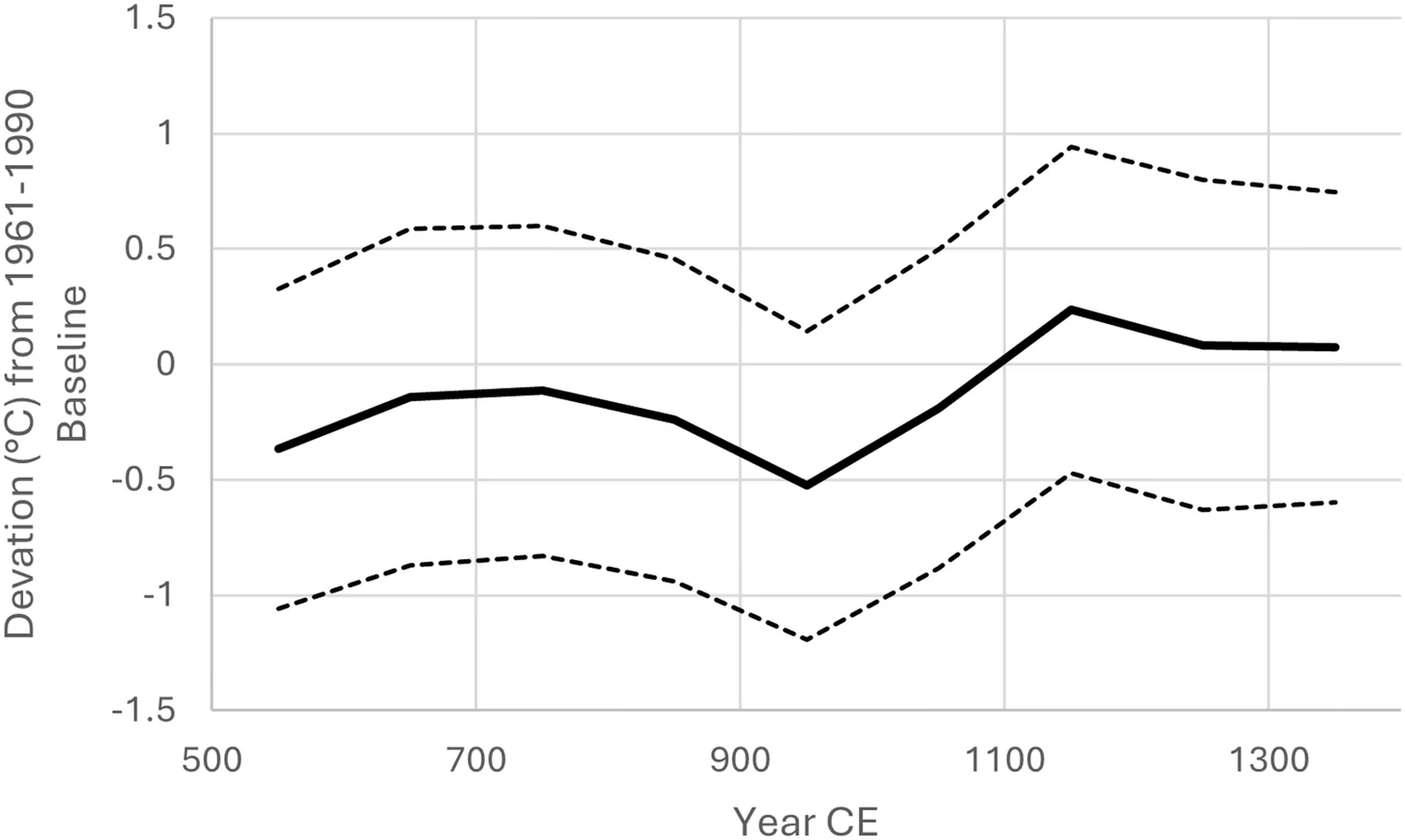
Temperature anomalies, by century, for the US Southwest, relative to the 1961–1990 CE baseline (=0) The dashed lines represent standard errors of the estimate derived from bootstrapping, see reference.^39^

The IPCC defines *climate change* as a change in the state of the climate that can be identified by changes in the mean and/or the variability of its properties, and that persists for an extended period, typically decades or longer. It may be due to natural internal processes or external forcings such as modulations of the solar cycles or volcanic eruptions, or from persistent anthropogenic changes in the composition of the atmosphere or in land use.^41^ The episode of elevated temperature from the early 1100s through the 1300s reflected in the pollen record constitutes a change in the state of the climate and was of sufficient magnitude and duration that people would have experienced it as permanent. There were also several multi-decade periods of reduced precipitation reflected in the tree-ring record that would have been experienced as a change in state of the climate. Thus, people of this society experienced, and were forced to adapt to, climate change.

The economy of ancestral Puebloan society can be characterized as having followed a domestic mode of production.^42^ In this economic system, the cultural ideal is for households or other small groups of relatives to produce their own food supply, including reserves as insurance against poor harvests in some years. Families settle adjacent to or directly on the lands that they cultivate, and farm sizes are tailored to the food needs of the family as opposed to how much that family could potentially produce.^43^^,^^44^ As a result, while there was a division of labor within households, and food could be exchanged for labor or other goods or given as gifts or loans to other households, very few people depended entirely on food produced by other households, and every household was engaged in food production to some extent.

Demographic patterns in this society are best understood within the Central Mesa Verde region study area of the Village Ecodynamics Project (see Figure 1), a long-term effort to understand human-environment relationships through archaeological data synthesis, paleoenvironmental reconstruction, and agent-based modeling.^38^^,^^45^ Ancestral Pueblo horticulturalists settled this 4,600 km^2^ area, which includes present day Mesa Verde National Park and Canyons of the Ancients National Monument, by around 600 CE, and from that time to 1260 CE, a period of roughly 30 human generations, an initial population of a few thousand settlers grew to number about 26,000 persons^30^; communities grew from a maximum size of about 80 persons (The Dillard Site Community) to a maximum of about 800 persons (Yellow Jacket Pueblo)^46^^,^^47^; and population densities, relative to lands on which maize could be grown, increased from about 2 persons per square kilometer to as high as 20 persons per square kilometer.^30^ Improvements in the efficiency of maize grinding and cooking also occurred over this period,^48^^,^^49^ and residents intensified production of domestic turkeys as a substitute for hunted game,^50^ but the division of labor above the household level was relatively constant, with little evidence of specialized production of manufactured goods.^51^

By the 1260s CE, the population density of the region was at an all-time high. Residents had already weathered significant long-term changes in annual precipitation and temperature over the centuries, but by the mid-1200s a large fraction of the population had been forced to settle lands that were suitable for agriculture at the time but were also vulnerable to drought. The situation is summarized in Figure 4, which displays the distribution of known settlements at 1265 CE, where the shading of the landscape reflects the fraction of years in the decade between 1265 and 1274 when there was sufficient heat and precipitation to produce a maize crop on that pixel, based on the PaleoCAR reconstruction.^31^ The resident populations of occupied settlements are based on a combined analysis of pottery assemblages, architectural traits, and building counts at these sites, with the unit of population being an extended household of six persons each.^30^ Some of the depicted settlements were founded long before 1265, but many were newly-founded around this time, including several large villages.^52^

**Figure 4:**
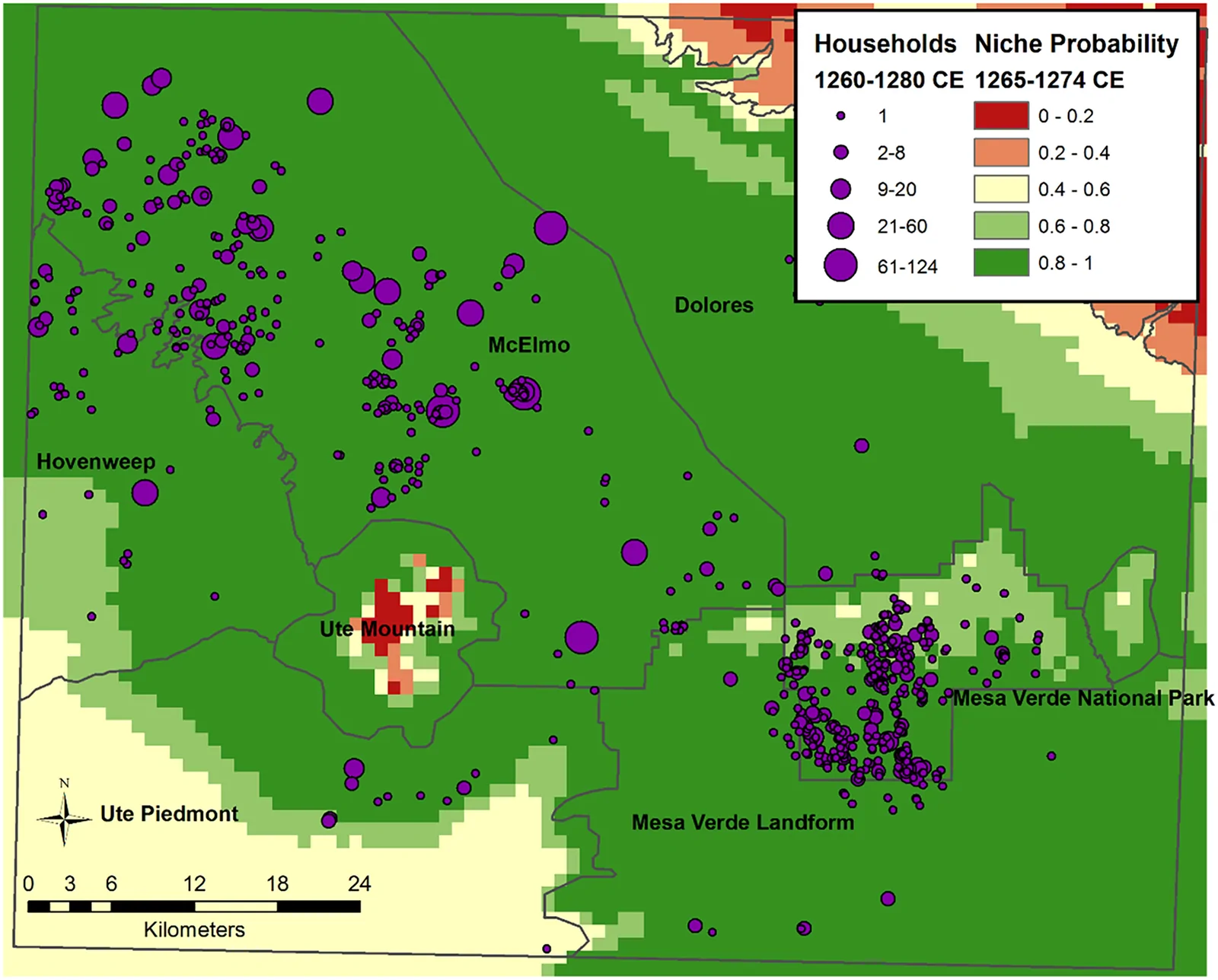
Distribution of Population and Niche Probabilities, 1265–1274 CE The niche probability is the fraction of years in the stated interval during which a location, modeled using 800-m pixels, received sufficient temperature and precipitation to produce a mature maize crop.

The situation appears to have been reasonably secure at this time, but in reality, the settlements in the southwestern half of the study area were on marginal lands that were vulnerable to decreases in precipitation. Residents of this southwestern half seem to have recognized and responded to this vulnerability by investing in community defensive works to guard against years when they might have more food than their neighbors.^53^ And when such a decrease set in, beginning in 1275, the results were catastrophic. The impact of this downturn is shown in Figure 5.

**Figure 5:**
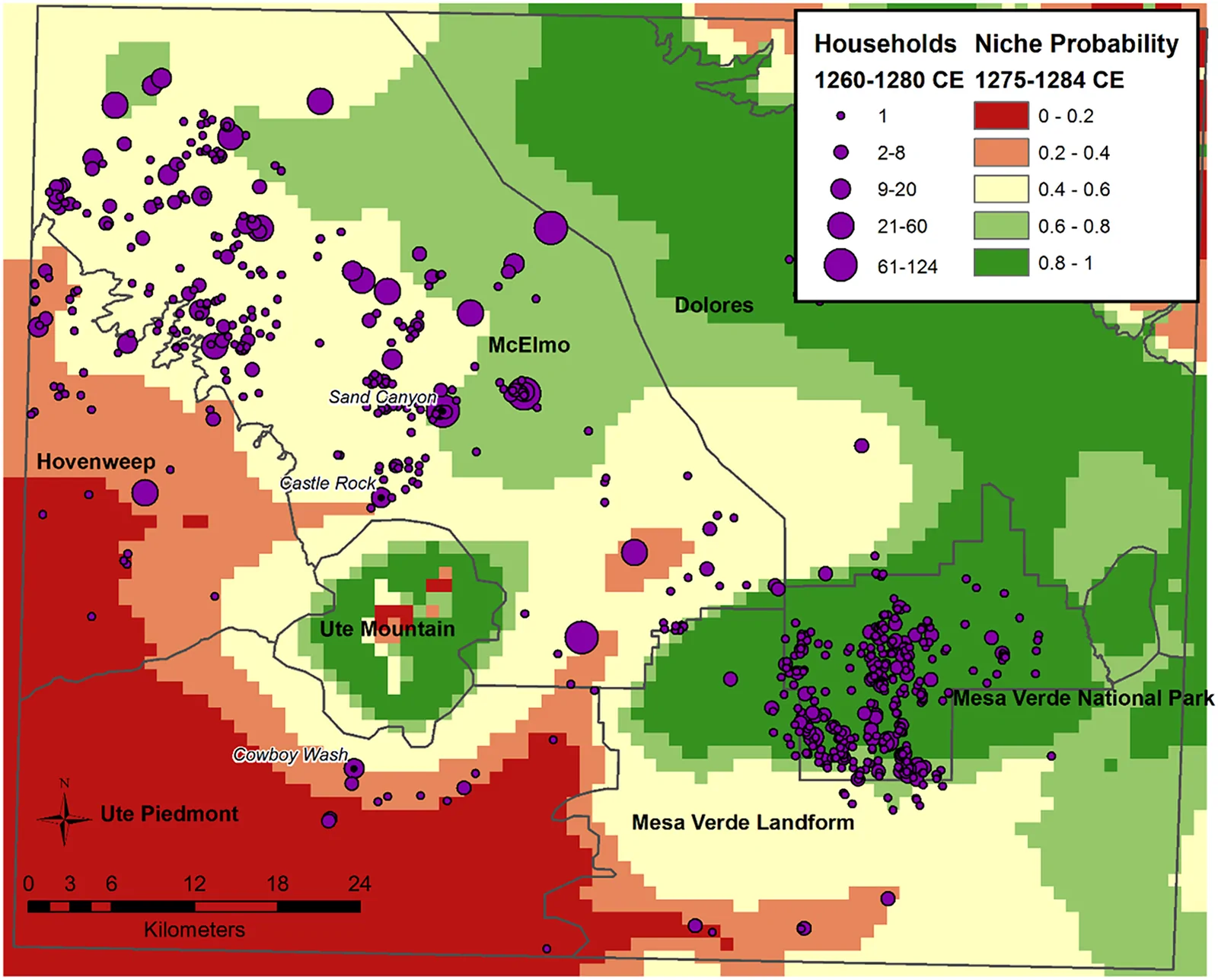
Distribution of Population and Niche Probabilities, 1275–1284 CE The Niche Probability is the fraction of years in the stated interval during which a location, modeled using 800-m pixels, received sufficient temperature and precipitation to produce a mature maize crop. Sites mentioned in the text are labeled.

It is important to note that initial studies suggested that the region could still have produced enough maize to feed the population during this period,^54^ but more recent gardening experiments suggest that paleo productivity reconstructions based on correlations between tree-ring series and weather station data under-estimate the true variance in annual production estimates.^55^ Regardless, as many as a third of the people living in the region at 1275 CE found themselves on land that produced an inadequate crop for long-term subsistence, and which could not produce any crop at all for at least five of the subsequent ten years. This would have depleted stored reserves to critical levels. Perhaps more importantly, the most productive lands were settled too densely for those residents to provision those who were hungry. For example, in Mesa Verde National Park, the population density at 1275 CE was approximately 15 p/km^2^, while only about 15 percent of the land had appropriate soils for agriculture.^30^^,^^56^ This area of arable land per person is close to the area needed for long-term subsistence in experimental gardening results (160 kg maize/person / a global average of 173 kg maize/ha).^55^ This means that, even if the requisite transport and distribution mechanisms had been in place, the resident population of the most productive lands could not have produced the necessary surplus. In this sense, disaster derived from the way longer-term climate change, which had increased the vulnerability of marginal land to drought, interacted with shorter-term weather variation and a settlement system that had taken shape in a cooler and wetter climate.

The resulting famine led to rapid relocation of the entire society. Excavations in settlements located in differentially affected regions give a sense of what unfolded. The Cowboy Wash community, located in the southwestern slopes of Ute Mountain, was so deeply affected that the residents simply walked away, leaving most of their personal belongings behind and their buildings intact.^57^ At Castle Rock Pueblo, in McElmo Canyon north of Ute Mountain, at least 40 residents were killed in a battle that ended the occupation of the village.^58^^,^^59^ And at Sand Canyon Pueblo, in a more productive area, some residents were killed in raids, many others burned their dwellings with their personal belongings inside before relocating, and the old or infirm stayed behind and lived out their lives by scavenging, hunting and gathering.^60^^,^^61^^,^^62^ It is also important to note that warriors were not the only people to suffer violent trauma during this period—osteological studies show that women and children were also common victims.^63^

A good way to perceive the tempo of collapse is to look at the date distribution of timbers harvested for new construction. Using the method of dendrochronology, researchers can build a barcode from annual growth rings on timbers preserved in archaeological sites. Using this bar code, analysts can cross-date timbers against the master chronology, such that one can determine the precise year in which a tree was harvested for construction when the outermost rings are preserved. A century of intensive sample collection combined with an abundance of preserved wood in dry cliff dwellings and burned subterranean buildings has produced a sample of thousands of such tree-ring cutting dates.^26^^,^^64^ The distribution of timbers for which the year of harvesting can be determined is given in Figure 6, with these dates aggregated into 5-year bins ranging from 1100 CE to 1300 CE. What this distribution shows is that, while the total number of dated timbers in each bin is due to a range of factors, including patterns in past cultural practices and building locations as well as biases in sampling, there is clear evidence for continuous timber harvesting for new construction for every 5-year bin between 1105 and 1280, including more than 100 dates for each 5-year bin between 1250 and 1275 CE. In addition, one can see a clear west-to-east progression in the cessation of timber harvesting, from about 1265 CE in Southeast Utah to 1275 CE in the Montezuma Valley (the location of Cowboy Wash, Castle Rock, and Sand Canyon pueblos), and 1280 CE on Mesa Verde itself (the location of Cliff Palace and Balcony House). In general, Southeast Utah was more arid than the Montezuma Valley, and the Montezuma Valley was more arid than Mesa Verde. So, the society was already struggling with climate changes that were underway prior to 1275 CE. The drought that was the proximal cause of relocation began in 1275 CE, but even during this period conditions remained adequate on Mesa Verde for new construction to continue. But as relocation proceeded, new construction stopped on Mesa Verde as well. Based on these data, it is highly unlikely that any ancestral Pueblo people remained in the Central Mesa Verde region by 1285 CE.^65^

**Figure 6:**
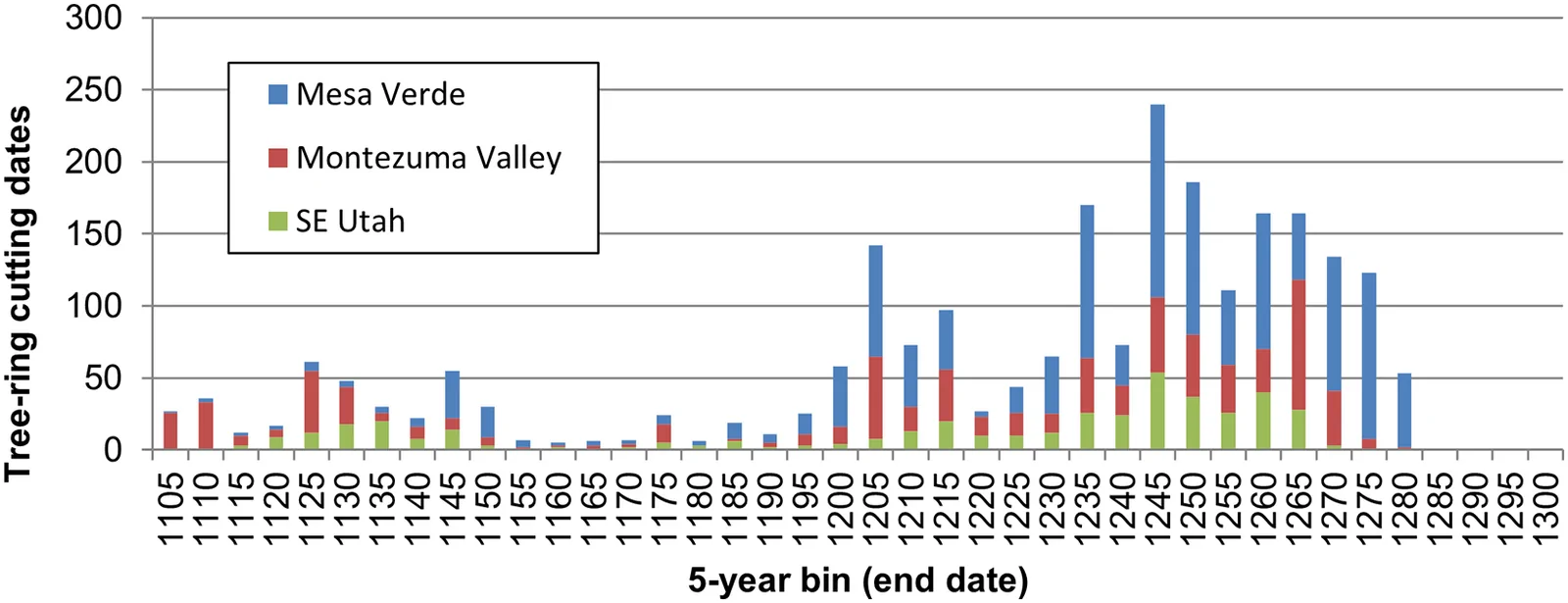
Histogram of Tree-Ring cutting Dates, in 5-year Bins

## The relocation process

Residents of the Central Mesa Verde region moved to several different locations in the late 1200s CE, but the majority moved about 400 km to the southeast, to the Tewa Basin in the Northern Rio Grande valley of New Mexico, also indicated in Figure 1. That this was the primary relocation destination is well supported by a range of evidence, including metric biological traits compiled from ancestral skeletal remains, ancient mitochondrial DNA (mtDNA) of domestic turkeys, patterns of obsidian exchange, correspondence in the timing and magnitude of population changes in the two regions, continuities in landscape ritual features, etymological traces of Mesa Verde region material culture in the present-day indigenous language of the destination area, and oral traditions among the speakers of this language.^20^^,^^29^^,^^66^^,^^67^^,^^68^^,^^69^^,^^70^^,^^71^^,^^72^ There are alternative views in the literature,^73^^,^^74^^,^^75^^,^^76^ but these focus on alternative or negative interpretations of the evidence and generally discount descendant community knowledge.^77^

Demographic reconstructions for the source and destination areas in Figure 1, based on settlement and associated pottery data, are presented in Figure 7.^78^ These show complementary changes in the pace of relocation, beginning in the middle decades of the 1200s CE and concluding by the 1290s. Population had been increasing in the destination area from the 1100s CE, but it is clear that population relocation from the Central Mesa Verde transitioned from a trickle to a flood after 1250 CE, with perhaps 15,000 people relocating in the 1270s. It is also clear from analyses of the size distribution of abandoned settlements in the Central Mesa Verde, and newly established settlements in the Tewa Basin, that the size of relocating groups increased as the process continued.^29^ Indeed, villages constructed in the Tewa Basin after 1275 CE consisted of apartment-style dwellings built using “ladder-type” construction, implying rapid, community-scale relocation.^69^^,^^79^^,^^80^^,^^81^

**Figure 7:**
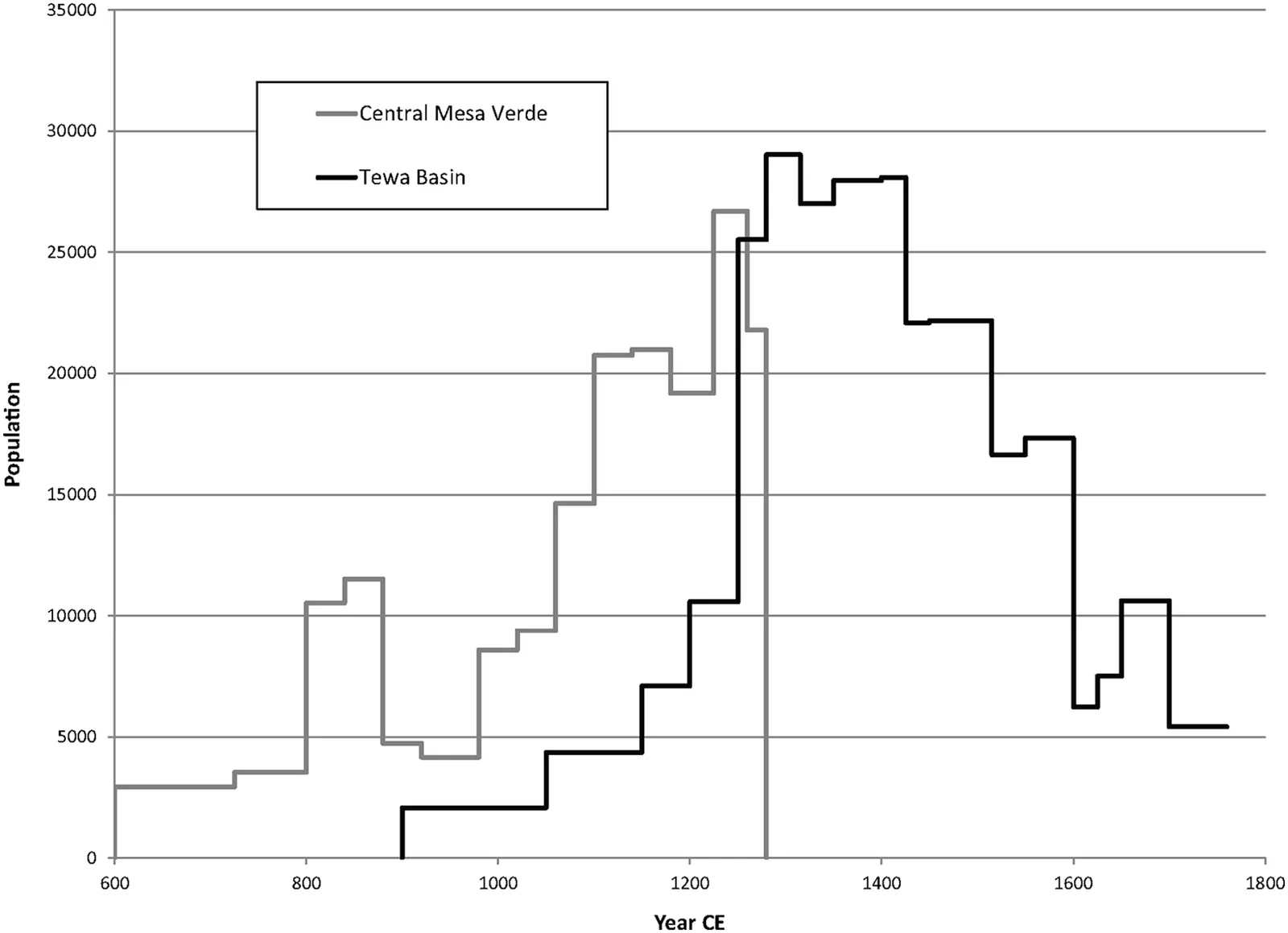
Population History of the Source and Destination Areas of the Relocation (after Ortman 2016: Figure 6)

The scale of governance in the ancestral Pueblo world is debated,^65^^,^^82^^,^^83^ but oral traditions in descendant Tewa communities of the destination area suggest that the final stages of the relocation process were organized and supported by leaders whose influence transcended individual communities. Individuals named in the published literature include “Long Sash,” a person who led Tewa ancestors on their migration and is memorialized today as the constellation Orion^84^; and “P’oseyemu,” a legendary figure who proposed relocation as an adaptive solution to society’s ills and who is honored to this day in annual mid-winter ceremonies.^68^^,^^85^^,^^86^ Oral traditions are post-hoc construals of events,^67^ so it is possible that social memories among living descendants overstate the importance and role of leadership in the relocation process. Nevertheless, there is no requirement for social memories to enshrine the names of acknowledged key figures who are still celebrated in song today.^86^ Archaeological studies have also demonstrated that relocation did not merely involve a change in residence locations, but also involved simultaneous cultural changes that have at times made it difficult for archaeologists to trace the relocation process.^29^^,^^35^^,^^87^ For these reasons, it seems most likely that the relocation process was not spontaneous but was supported by political leaders of the time.

Several lines of evidence suggest the relocation process was not peaceful and involved some level of coercion. Studies of human skeletal remains indicate a marked uptick in the incidence of perimortem trauma, especially skull and forearm fractures and embedded projectile points, in Central Mesa Verde settlements occupied into the 1270s CE.^63^^,^^88^ As settlements were vacated, an increasing fraction of residences were intentionally destroyed with household belongings arranged inside, suggesting a moralizing discourse regarding the reasons relocation was necessary.^29^ Finally, characteristic elements of Central Mesa Verde material culture are conspicuously absent in the new settlements of the Tewa Basin relocation area, reinforcing the interpretation of a strong moralizing discourse against the former homeland and its traditions.^29^ While not all people chose to relocate, as the process unfolded, those who were left behind suffered marked declines in living standards, including reversion to hunting and gathering, residence in unmaintained buildings, and high risk of interpersonal violence.^62^^,^^89^ These post-apocalyptic conditions were terminal for those who remained. In short, the evidence suggests it took significant political leadership and perhaps even coercion for community-scale relocations to occur, even in the face of famine and widespread violence. This is a sobering thought for the present.

There is evidence from the destination area that relocation was driven, at least in part, by a desire to reduce risks from climate-related hazards the society had experienced in the Central Mesa Verde. A significant fraction initially relocated to upland areas where precipitation-based farming was possible, while others settled along major rivers and their tributaries where floodwater farming and diversion of surface flows was feasible.^90^^,^^91^^,^^92^ Over time the population increasingly concentrated in the lowlands and focused on diversion of runoff and surface stream flows.^92^^,^^93^^,^^94^^,^^95^^,^^96^^,^^97^^,^^98^ These strategies were less vulnerable to climate variation because they effectively averaged and concentrated precipitation across larger areas and longer periods of time. That these strategies were in fact more sustainable and resilient is reflected in the fact that Tewa Basin settlements were both larger and longer lived on average than Central Mesa Verde settlements. Indeed, several Tewa communities established around the time of relocation are still inhabited today.

There is, however, one very important feature of this episode of relocation that must be emphasized—as the population of the source area declined, climate-related hazards would have also declined because more of the best-watered and most reliable lands for agriculture would have become available. Indeed, at least some residents could have continued to live in the source area during the extended period of reduced precipitation and increased temperature. Nevertheless, the entire population relocated permanently and did not return when climate conditions became more advantageous. This feature suggests the social forces unleashed by climate change amplify climate-related drivers as relocation proceeds. Indeed, the magnitude of change in ancestral Pueblo society that took place during the 1200s CE far exceeded the direct impacts of the climate changes the population experienced, likely due to the chaotic effects of these perturbations, and initial relocations, for the remaining human networks.^99^

## Relocation consequences

What were the consequences of relocation for living standards? One way of assessing such changes comes from accumulation rates of broken pottery vessels. Because all individuals need a consistent supply of food, accumulations of cooking potsherds are consistent per capita and per unit time and exhibit low income elasticity.^100^ Decorated serving vessels, in contrast, reflect investment in socializing and etiquette, and one would thus expect their accumulation rates to increase with household incomes.^101^ The ratio of service wares to cooking wares in trash assemblages thus provides a proxy for changing incomes per capita and per unit time. These data are shown in Figure 8. The ratio shows long-term improvement as central Mesa Verde society developed, until the middle decades of the 1200s CE, when living standards declined in the run-up to relocation. In the destination area, in contrast, living standards did not match those of the pre-relocation area until after 1350 CE, several generations after relocation. However, after 1450 CE the economy clearly improved, such that unprecedented living standards were realized in the century prior to arrival of the Spanish conquistadors.

**Figure 8:**
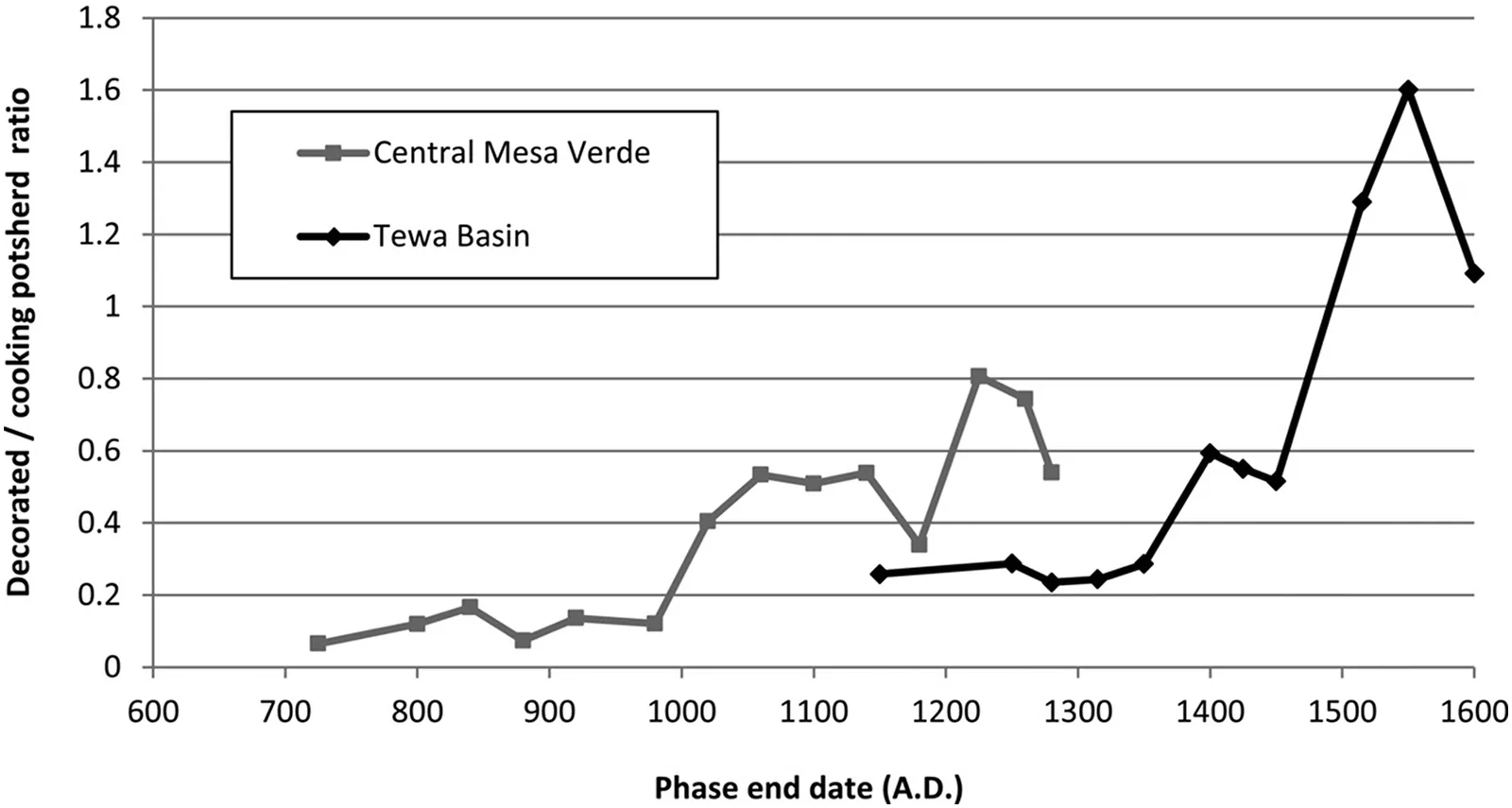
Ratio of Decorated / Cooking Potsherds Over Time. Points are plotted at the end date of an interval (after Ortman 2016: Figure 5.7)

Another way to look at changing living standards is through changes in agglomeration. Ever since Adam Smith’s *The Wealth of Nations,*^102^ economists have recognized that population agglomeration, even in the absence of technological change, is associated with economic growth through an expansion in market size and the associated division of labor.^103^^,^^104^^,^^105^ The fraction of population living in settlements above a certain size is thus a useful, if conservative, index of economic development. These data are presented in Figure 9, which shows the fraction of population living in villages of at least fifty persons in the Central Mesa Verde, and the fraction of population living in towns of at least five hundred persons in the Tewa Basin, over time. These data show very low levels of agglomeration in the Central Mesa Verde, followed by a substantial and sustained increase following relocation to the Tewa Basin. Other studies show significant increases in specialized production and inter-regional exchange also accompanied this extended episode of agglomeration.^106^^,^^107^^,^^108^^,^^109^^,^^110^

**Figure 9:**
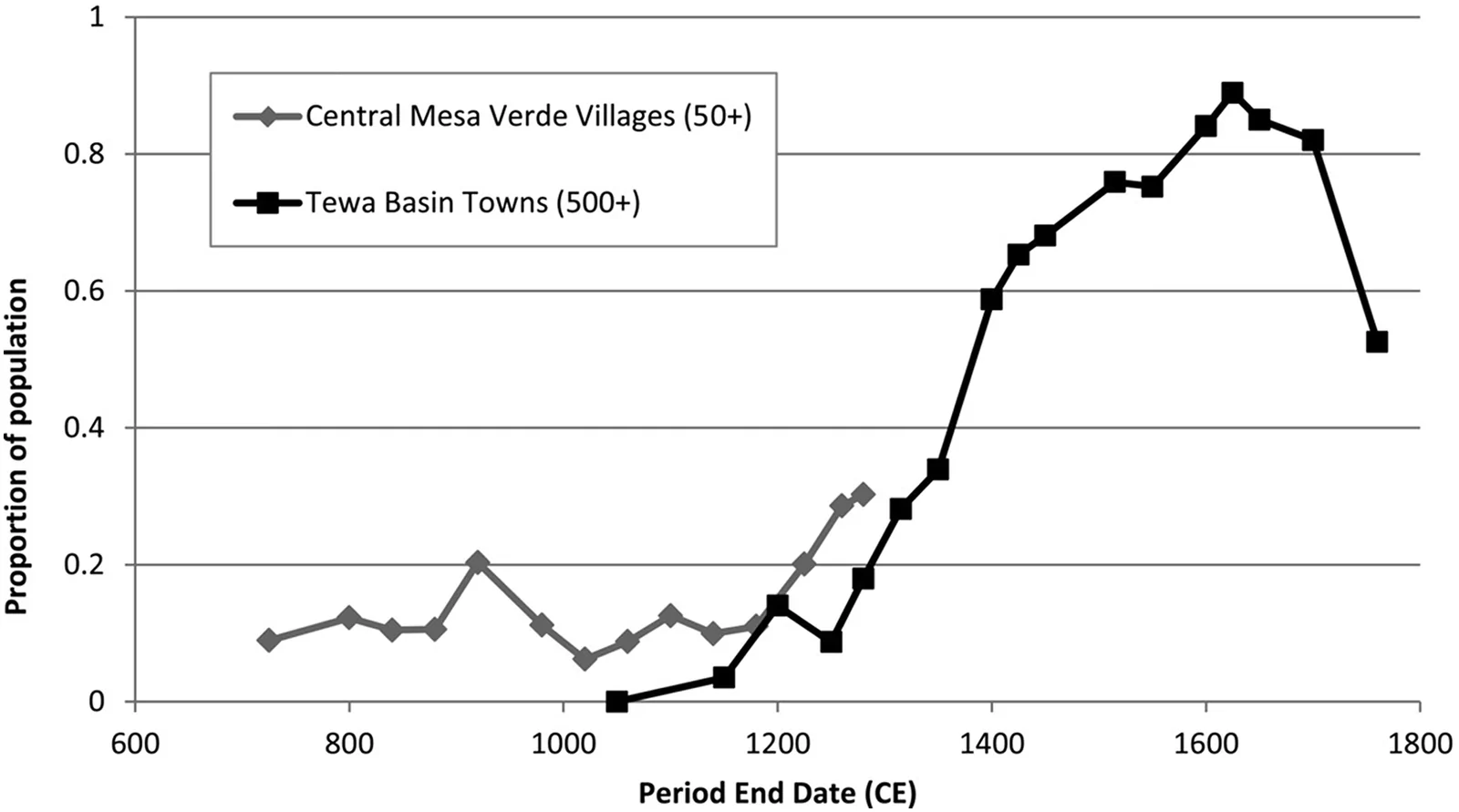
Agglomeration Rates Over Time (after Ortman 2016: Figure 5.5)

A final way to assess the impact of relocation for living standards is by tracking rates of interpersonal violence. Figure 10 summarizes the fraction of individuals on which direct evidence of interpersonal violence, either antemortem or perimortem, is apparent in the skeleton. Once again, data points reflect the end date of the interval. The extreme peak in Central Mesa Verde during the late 1100s CE is likely an artifact of sampling and taphonomic bias, but the overall time series are based on information from over 600 individuals in each region,^88^ and they clearly show that interpersonal violence was much less prevalent following relocation than it had often been in the Central Mesa Verde, from the relocation period onward.

**Figure 10:**
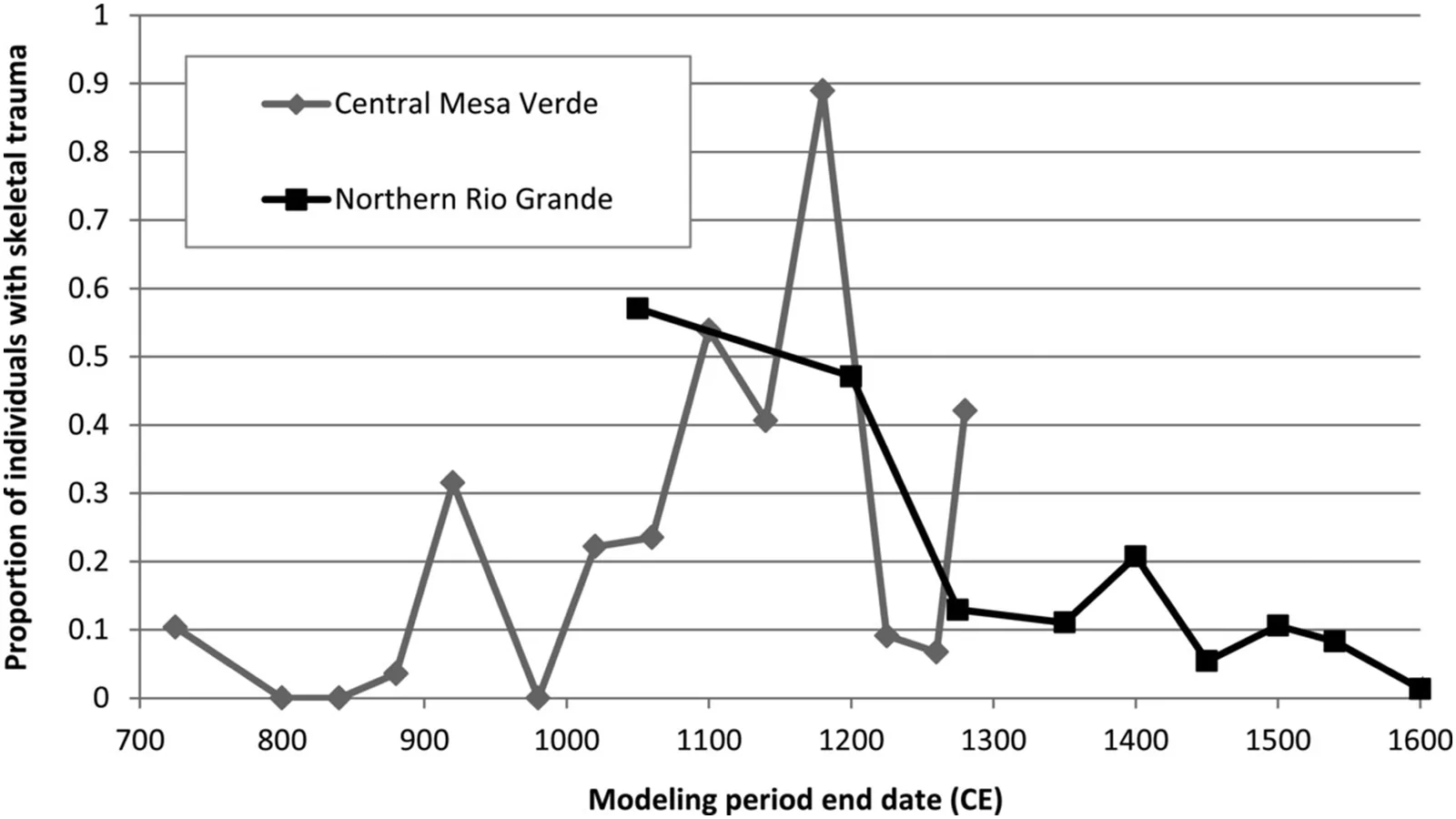
Interpersonal Violence Rates Over Time (after Ortman 2016: Figure 5.4)

These various lines of evidence show that the consequences of relocation for the population were complex and not realized all at once. Interpersonal violence and agglomeration improved as relocation took place, but it took several generations for ancestral Pueblo society to reclaim the level of economic development it had enjoyed in its prior location. At the same time, economic conditions continued to improve in the generations following relocation. Several lines of evidence, including Tewa oral histories, indicate that substantial institutional changes accompanied relocation,^34^^,^^111^^,^^112^ so it is not possible to associate the consequences of relocation directly with reductions in climate-related hazards. In fact, it is exceedingly difficult to attribute patterns of change in any society to a single factor due to the inherent nature of human societies as complex systems. What is perhaps reasonable to say is that at least some of the changes that occurred in the generations following relocation were not envisaged at the time of relocation, but they emerged as a byproduct of other changes that accompanied relocation. Regardless of causality attributions, it is possible to say that the overall, long-term consequences of relocation for this society were positive and included a reduction in climate-related hazards. So, it is possible to state that, despite negative impacts for the people who actually moved, the relocation process described here was positive and adaptive in the big picture and over the long term.

## Discussion

The settlements considered in this paper were different from contemporary urban settlements in many ways. They were generally very small, containing only a few hundred to a few thousand people at most. Their food supplies were generated almost entirely locally, by the residents, and within a few kilometers of the settlement itself. Their economies were much more closely tied to changes in local climatic conditions than is the case for contemporary urban areas. Their technology and energy sources were rudimentary by contemporary standards, and as a result their capacity to adapt through technological innovation was very limited. Finally, there was no regional government to orchestrate adaptive responses or allocate regional surpluses to human needs.

At the same time, these settlements were populated by human beings with the same abilities and needs as residents of contemporary cities. There were elders, parents and children who needed to make decisions to sustain their families in the future. Despite being much smaller, the settlements and the society in which these people lived were complex systems of social and infrastructural networks through which goods, energy and information flowed. Finally, the settlements and the settlement system in which these people lived exhibited the same aggregate properties and nonlinear dynamics that are observed in contemporary urban systems, including central-place functional and institutional hierarchies,^32^ Zipfian rank-size distributions,^113^^,^^114^ and typical urban scaling elasticities with regard to density, productivity, and the division of labor.^7^^,^^115^ So the differences between Ancestral Pueblo society and contemporary urban systems are more a matter of degree than kind.^8^^,^^9^^,^^101^ Thus, the degree to which the dynamics observed in this case of relocation are relevant for the present is not ruled out by differences in scale and development; it is rather a question for further investigation.

A summary of the episode of climate-driven population relocation discussed here is as follows. Between 600 and 1260 CE, a society of maize horticulturalists took root in the northern drainages of the San Juan River, in what is today Colorado and Utah, USA. This society successfully weathered several extended periods of increased temperature and dryness over the centuries, but it was generally successful, such that the population, population density, and size of communities all grew by an order of magnitude over this period. However, the basic principles of economic and social organization did not change as much, and as the population grew an increasing fraction of the people were forced to settle on lands that were vulnerable to climate change. When the next extended period of warmer and drier conditions set in, about 30 percent of the population found itself on land that could no longer support them, and the remainder of the society did not have the capacity to produce and distribute the necessary surpluses to make up the difference.

The entire society collapsed rapidly as a result, and community-scale relocation became the dominant adaptive response. Emergent populist leadership, moralizing discourses regarding the failed society, and significant social conflict and violence accompanied this process. A significant fraction of the population moved about 400 km to the southeast, to an area where more reliable floodwater and stream diversion farming was possible. It took several generations for living standards to recover and surpass those that had existed prior to relocation, but eventually the relocated settlements did become larger, more productive, more prosperous, and safer than those of the pre-relocation society.

This episode suggests several features of relocation as an adaptive response to climate change. This specific case suggests that:

• entire human communities have indeed relocated in the face of climate-related hazards in the past;
• relocation can occur within a span of decades or even years;
• even in a small-scale society, relocation is a political process that requires strong leadership;
• relocation can generate its own momentum as it proceeds;
• even in a small-scale society with limited capital accumulation, relocation may be conflict-ridden and not entirely voluntary;
• the social consequences of climate change for human systems can far exceed the direct impact of climate changes themselves;
• the short-term consequences of relocation are not necessarily positive;
• longer-term consequences can be positive but are not necessarily attributable to reductions in climate-related hazards; and
• relocation can provide an opportunity for rapid social and institutional change that solves other problems and facilitates longer-term adaptation.

Whether these characteristics are generally useful for climate mitigation-related efforts in the present will depend on how commonly these characteristics are associated with other episodes of climate-related relocation. There is a need for additional studies of such episodes from an interdisciplinary perspective like the one adopted here, from as broad a range of human societies and contexts as possible, to aid governments who may need to make relocation decisions in coming decades. In this process, it is important for specialists to be attentive to questions being asked by the climate change adaptation community as opposed to questions that animate specific research communities in archaeology or history. It is also important for specialists to be judicious and intellectually honest regarding what is well-established vs. hypothetical, and regarding the broader implications of specific cases.

## Acknowledgments

I thank Jose Lobo, Michael Smith, Dan Lawrence, and Chris Roberts for discussions, and two helpful reviewers for their comments on earlier versions of this paper.

## Declaration of interests

The authors declare no competing interests.
